# LncRNA FEZF1-AS1 Promotes Multi-Drug Resistance of Gastric Cancer Cells via Upregulating ATG5

**DOI:** 10.3389/fcell.2021.749129

**Published:** 2021-11-01

**Authors:** Zhifu Gui, Zhenguo Zhao, Qi Sun, Guoyi Shao, Jianming Huang, Wei Zhao, Yuting Kuang

**Affiliations:** ^1^Department of General Surgery, The First Affiliated Hospital of Soochow University, Suzhou, China; ^2^Department of General Surgery, Jiangyin Hospital Affiliated to Medical College of Southeast University, Wuxi, China; ^3^Department of Biomedical Sciences, City University of Hong Kong, Kowloon, Hong Kong, SAR, China

**Keywords:** FEZF1-AS1, gastric cancer, multi-drug resistance, ATG5, autophagy

## Abstract

Long non-coding RNAs (lncRNAs) play important roles in human cancers including gastric cancer (GC). Dysregulation of lncRNAs is involved in a variety of pathological activities associated with gastric cancer progression and chemo-resistance. However, the role and molecular mechanisms of FEZF1-AS1 in chemoresistance of GC remain unknown. In this study, we aimed to determine the role of FEZF1-AS1 in chemoresistance of GC. The level of FEZF1-AS1 in GC tissues and GC cell lines was assessed by qRT-PCR. Our results showed that the expression of FEZF1-AS1 was higher in gastric cancer tissues than in adjacent normal tissues. Multivariate analysis identified that high level of FEZF1-AS1 is an independent predictor for poor overall survival. Increased FEZF1-AS1 expression promoted gastric cancer cell proliferation *in vitro*. Additionally, FEZF1-AS1 was upregulated in chemo-resistant GC tissues. The regulatory effect of FEZF1-AS1 on multi-drug resistance (MDR) in GC cells and the underlying mechanism was investigated. It was found that increased FEZF1-AS1 expression promoted chemo-resistance of GC cells. Molecular interactions were determined by RNA immunoprecipitation (RIP) and the results showed that FEZF1-AS1 regulated chemo-resistance of GC cells through modulating autophagy by directly targeting ATG5. The proliferation and autophagy of GC cells promoted by overexpression of LncFEZF1-AS1 was suppressed when ATG5 was knocked down. Moreover, knockdown of FEZF1-AS1 inhibited tumor growth and increased 5-FU sensitivity in GC cells *in vivo*. Taken together, this study revealed that the FEZF1-AS1/ATG5 axis regulates MDR of GC cells via modulating autophagy.

## Introduction

Gastric cancer (GC) is the fourth most frequent cancer which cause the third cancer-related mortality worldwide (Sung et al., 2021). Currently, surgical intervention and chemotherapy or radiotherapy are the main treatment modalities for gastric cancer (Asaka et al., 2001; McLean and El-Omar, 2014). Among the chemotherapeutic drugs that are applied in GC treatment, 5-fluorouracil (5-FU) and cisplatin-based chemotherapy are the commonly used ones (Sakuramoto et al., 2007; Lee et al., 2021). Although the advances of chemotherapy during the past decades have greatly improved the survival of patients, GC remains a major global public health problem because of the development of multi-drug resistance (MDR) (Orditura et al., 2014; Wu et al., 2016). MDR is a key barrier of GC treatment, resulting in therapeutic failure and cancer-related death (Wu et al., 2016; Luo et al., 2017). However, the underlying molecular mechanisms leading to MDR have not been fully elucidated (Chen et al., 2020). Therefore, understanding the underlying molecular mechanisms of chemoresistance and finding potential targets to provide new strategies for therapeutic approaches is an urgent clinical necessity.

Long non-coding RNAs (lncRNAs) are non-protein coding RNAs, that are longer than 200 nucleotides and they usually account for more than 80% of the transcripts of the entire genome of cells (Zhang et al., 2015; Luo et al., 2019). Several recent studies reported that dysregulated lncRNAs are involved in MDR in some cancers including GC through regulating mRNA transcription, translation, protein stability and so on (Yuan et al., 2016; Zhang et al., 2017). For example, overexpression of lncRNA PVT1 in gastric cancer cells promotes the development of MDR (Zhang et al., 2015). LncRNA UCA1 increases MDR of GC by downregulating miR-27b (Cheng et al., 2021). LncRNA MRUL promotes ABCB1 expression in MDR gastric cancer cells and MRUL depletion can reduce ABCB1 mRNA levels and reverse the MDR phenotype of cells (Wang et al., 2014). LncRNA NEAT1 may act as a miR-98-5p sponge to upregulate EGCG-induced CTR1 and promote cisplatin sensitivity in non-small cell lung cancer (Jiang et al., 2016). Autophagy is one of the molecular mechanisms of chemoresistance in several cancers including GC (Li et al., 2019; Xu et al., 2020; Ashrafizadeh et al., 2021). Recently, some studies had found that lncRNAs mediated chemoresistance of cancer cells through modulating autophagy. For example, Yuan et al. (2016) reported that HIF-2a-MALAT1-miR-216b axis regulates multi-drug resistance of hepatocellular carcinoma cells via modulating autophagy. LncRNA EIF3J-DT induces chemoresistance of gastric cancer via autophagy activation (Luo et al., 2021). LncRNA GBCDRlnc1 induces chemoresistance of gallbladder cancer cells by activating autophagy (Cai et al., 2019). These studies suggest that activation of autophagy by lncRNAs play an important role in chemoresistance of cancer cells.

The lncRNA FEZ family zinc finger 1 antisense RNA 1 (FEZF1-AS1) is located on the opposite strand of gene FEZF1 and mapped to chromosome 7. FEZF1-AS1 encodes a 2,564 bp transcript (Wu et al., 2017). As described in previously studies (Bian et al., 2018), lncRNA FEZF1-AS1 was upregulated in primary colorectal carcinoma (CRC), non-small cell lung cancer (NSCLC) as well as GC tissues and cells, and the overexpression of ncRNA FEZF1-AS1 was correlated with poor prognosis. Dysregulation of lncRNA FEZF1-AS1 promoted the proliferation and migration of CRC (Bian et al., 2018), NSCLC (Huang et al., 2020), and GC cells (Hui et al., 2020). Furthermore, knock down of lncRNA FEZF1-AS1 significantly suppressed the proliferation and invasion of tumor cells (Chen et al., 2016; Bian et al., 2018). Moreover, recent studies showed that upregulation of lncRNA FEZF1-AS1 promoted GC cell proliferation and indicated a poor prognosis of gastric cancer (Wu et al., 2017). Although FEZF1-AS1 expression is known to be associated with poor prognosis in several types of cancers including GC (Wu et al., 2017; Zhou et al., 2019; Chen et al., 2021), its role in MDR of GC cells remains unknown.

In this study, we investigated the association between FEZF1-AS1 and ATG5 in GC cells, and the regulatory effect of FEZF1-AS1 on MDR and its possible underlying mechanism.

## Materials and Methods

### Patients and Tissue Samples Collection

A total of 94 matched gastric cancer (GC) tissues and adjacent non-tumor tissues were acquired from the Affiliated Jiangyin Hospital of Southeast University Medical College (Wuxi, Jiangsu province, China) from May 2014 to July 2018. Patients (*n* = 40) received no treatment of radiotherapy or chemotherapy prior to surgery. The tumors were defined as chemo-resistant (*n* = 27) or chemo-sensitive (*n* = 27) to chemotherapy with 5-FU according to the response evaluation criteria of solid tumors before surgery. GC tissues and the adjacent normal tissues were obtained from these patients after surgery and were immediately frozen in liquid nitrogen and stored at −80°C for further analysis. The patients involved in this study were 56 males and 38 females with age ranging from 45 to 82 years old. Informed consent was obtained from each participant and the study was approved by the ethics committees of Affiliated Jiangyin Hospital of Southeast University Medical College (No. JYCP13W11).

### Cell Culture and Treatment

Gastric cancer (GC) cell lines including MKN-49P, MGC-803, BGC-823, SGC-7901, and NCI-N87 cells, and the normal human gastric epithelial cell line (GES-1) were obtained from the American Type Culture Collection (United States). Cells were cultured in RPMI-1640 medium (Sigma, United States) supplemented with 10% (v/v) fetal bovine serum (Gibco, United States), penicillin sodium (100 U/ml) and streptomycin (100 μg/ml) in a humidified atmosphere containing 5% CO_2_ at 37°C. To construct the drug-resistant GC cell subline, SGC7901 cells were cultured and exposed to a gradually increasing concentration of 5-FU or cisplatin (CDDP) (Sigma-Aldrich, St. Louis, MO, United States) from 5 to 30 μM. Cell viability was measured by Cell Counting Kit-8 (CCK-8, Abcam).

### Cell Transfection and Lentivirus Infection

The FEZF1-AS1 and ATG5 CDS sequence were amplified and cloned into the multiple cloning site (MCS) of the pcDNA3.1 vector or PRK5 vector (Invitrogen, Shanghai, China). Small interfering RNA (siRNA) targeting FEZF1-AS1 (5′-GGGTTTCTGCAGGAACTTTGA-3′, 5′-GCACAAGATCATT CACACGCA-3′), ATG5 (5′-GACAAGAAGACATTAGTGA GA-3′, 5′-GGAAACACCTCTGCAGTGGC-3′) and a negative control siRNA were projected and synthesized by Ribobio Co. (Guangzhou, China). Lipofectamine 3000 (Invitrogen, United States) was used to transfect siRNA or plasmids into cells according to the manufacturer’s instructions. Cells were incubated for 48 h, and then harvested for further analyses.

For stably knockdown of FEZF1-AS1 by lentivirus infection, the validated shRNA sequence of FEZF1-AS1 or negative control was synthesized and cloned into the pSIH-H1 shRNA cloning and expression lentivector. The lentiviral particles for infection were produced in 293FT (Thermo Fisher) and the cells were infected with lentivirus. The infected cell population was selected with puromycin, and knockdown efficiency of FEZF1-AS1 was determined by qRT-PCR.

### Quantitative Real-Time Polymerase Chain Reaction Analysis

Total RNA was isolated from tissues or cell samples by Trizol reagent (Invitrogen, United States) according to the manufacturer’s instructions. Then the total RNA was reversely transcribed into cDNA using the HiScript II RT Reagent Kit (Vazyme, China) according to the manufacturer’s instructions. RT-qPCR was performed using SYBR Green PCR Master Mix on an Applied Biosystems (ABI) StepOne Plus Sequence Detection System (Applied Biosystems, United States) in 96-well plates. β-actin was used as a reference control. The sequence of primers (Invitrogen, United States) was listed in Table 1. The Ct value for each sample was calculated and the results were calculated and expressed as 2^–ΔΔ^*^*C*^*^t^ as described in a previous study (Schefe et al., 2006).

**TABLE 1:** Sequences of primers used in this study.

**Gene name**	**Primer sequences (5′–3′)**
FEZF1-AS1	F: TTAGGAGGCTTGTTCTGTGT R: GCGCAGGTACTTAAGAAAGA
p21	F: AGGTGGACCTGGAGACTCTCAG R: TCCTCTTGGAGAAGATCAGCCG
Cyclin D1	F: TCTACACCGACAACTCCATCCG R: TCTGGCATTTTGGAGAGGAAGTG
ATG5	F: GCAGATGGACAGTTGCACACAC R: GAGGTGTTTCCAACATTGGCTCA
β-Actin	F: CATGTACGTTGCTATCCAGGC R: CTCCTTAATGTCACGCACGAT

### Flow Cytometric Analysis

To analyze the cell cycle, the transfected cells were harvested and stained with propidium iodide staining kits (KeyGen BioTech, Nanjing, China) according to manufacturer’s instructions. Cells were analyzed by FACSCalibur instrument (BD Biosciences, United States). For apoptosis analysis, the cells were washed with cold PBS after trypsinization, then suspended in 1× binding buffer prior to incubation with 5 μl of APC Annexin V and 5 μl of propidium iodide (PI) solutions, according to the manufacturer’s protocol for the APC Annexin V Apoptosis Detection Kit (BD Biosciences, United States). The cells were gently vortexed and incubated for 15 min at room temperature in the dark. The cells were analyzed using a FACS Aria flow cytometer (BD Biosciences, United States).

### Western Blotting

Total proteins were extracted from tumor tissues or cells using RIPA buffer (Beyotime, China) with protease inhibitors (Roche, Germany). Then the protein concentration was detected using a BCA Protein Assay Kit (Beyotime, China). Proteins (30 μg) were separated by 10% SDS-PAGE and transferred to polyvinylidene difluoride (PVDF) membrane. Membranes were blocked with 5% non-fat milk in TBST for 1 h at room temperature and incubated overnight at 4°C with the first antibodies against drug resistance-associated proteins MDR1 (1:2000, PAB30805, Bioswamp), MRP1 (1:1000, PAB33537, Bioswamp), ATG5 (1:1000, 10181-2-AP, Proteintech), β-actin (1:1000, 66009-1-Ig, Proteintech), Ubiquitin (1:1000, 3936, CST), LC3B (1:1000, 3836, CST), BCL2 (1:1000, 12789-1-AP, Proteintech), BAX (1:1000, 50599-2-Ig, Proteintech) and Cleaved caspase3 (1:1000, 9661, CST). The next day, the membranes were washed with TBST for three times and incubated with HRP-conjugated anti-IgG secondary antibody at room temperature for 1 h. Protein bands were visualized by using an Enhanced Chemiluminescence (ECL) Detection System (Tanon, China) according to manufacturer’s instructions.

### Cell Proliferation Assay

The proliferation ability of GC cells was measured using Cell Counting Kit-8 (CCK8) assay. Briefly, 1 × 10^4^ GC cells were seeded into 96-well plates with or without treatment as indicated. For CCK8 analysis,10 μl CCK-8 solution (Dojindo Laboratories, Japan) was added to each well. Then, the cells were incubated at 37°C for 2–4 h. The light absorbance at 450 nm was measured using an enzyme-labeling instrument (Thermo, United States). The OD values of each group were calculated by using GraphPad Prism 6 software (San Diego, United States). All experiments were repeated in triplicate.

### RNA Immunoprecipitation Assay

The Magna RIP RNA-Binding Protein Immunoprecipitation Kit (Millipore) was purchased and used to perform RNA Immunoprecipitation (RIP) analysis as previously described (Bian et al., 2018). Briefly, 1 × 10^7^ GC cells were harvested and lysed with lysis buffer. Total cell extracts were co-immunoprecipitated with anti-ATG5 antibodies (Proteintech), and the retrieved RNA was subjected to qRT-PCR analysis using specific primers of FEZF1-AS1. Total RNA (input controls) and normal rabbit IgG controls were assayed simultaneously to confirm that the detected signals were from the RNA specifically binding to ATG5.

### *In vivo* Ubiquitination Assay

Gastric cancer cells were transfected with HA-ATG5, Flag-Ub, or si-FEZF1-AS1 and treated with MG132 (proteasome inhibitor, 20 μmol/L) for 6 h. The ubiquitinylated ATG5 was measured by Western blot using an anti-Ub antibody following the immunoprecipitation of HA-ATG5 with an anti-HA antibody (51064-2-AP, Proteintech).

### Immunofluorescence

Cells were cultured on 24-well chamber slides and co-transfected with FEZF1-AS1 overexpression plasmids and si-ATG5 for 48 h. At the time of harvest, cells were washed with PBS and fixed with 4% paraformaldehyde for 15 min and then permeabilized with 0.01% Triton X-100 for 30 min. Then cells were stained with anti-LC3B antibody (Proteintech) overnight at 4°C. The next day, the slides were washed with PBS for three times and incubated with Goat anti-Rabbit IgG (H + L) Cross-Adsorbed Secondary Antibody, Alexa Fluor 488 (Thermo Fisher) for 1h at 37°C avoid light. In addition, all samples were treated with DAPI for nuclear staining. For confocal microscopy, Carl Zeiss LSM710 confocal microscope was used.

### Tumor Xenografts in Nude Mice

For tumor xenografts analysis, 32 BALB/c nude male mice (6- to 8-week-old, purchased from the Gempharmatech Company, China) were subcutaneously injected with 10^7^ sh-NC-SGC7901 or sh-FEZF1-AS1-SGC7901 cells. When the tumor volumes reached 100–150 mm^3^ after 7 days, nude mice were randomly subdivided into four groups with 8 mice in each group. Experimental groups were treated with 5-FU intraperitoneally (50 mg/kg) once every 2 days for 3 weeks, while control groups were treated with PBS. After 3 weeks, nude mice were sacrificed by cervical dislocation, and xenograft tumors were harvested and weighed. The tumor volumes of xenograft were measured by a caliper and calculated as V = 1/2 × (length × width 2). All animal experiments were approved by the ethics committees of Affiliated Jiangyin Hospital of Southeast University Medical College, and performed in accordance with the Institutional Committee for Animal Research and national guidelines for the care and use of laboratory animals.

### Statistical Analysis

All results are presented as mean ± standard deviation (SD). One-way/two-way analysis of variance (ANOVA) or Student’s *t*-test were used to compare the difference among different groups. The Kaplan–Meier method and log-rank test were applied to determine the difference in survival rates between two groups. GraphPad Prism 6.0 was used to draw all the plots. All statistical analyses were carried out using SPSS 19.0 software (IBM Corp., Armonk, NY, United States). *P*-values < 0.05 were considered statistically significant.

## Results

### LncFEZF1-AS1 Was Overexpressed in Gastric Cancer Tissues and Cell Lines, and Correlated With Poor Prognosis of Gastric Cancer Patients

**FIGURE 1:**
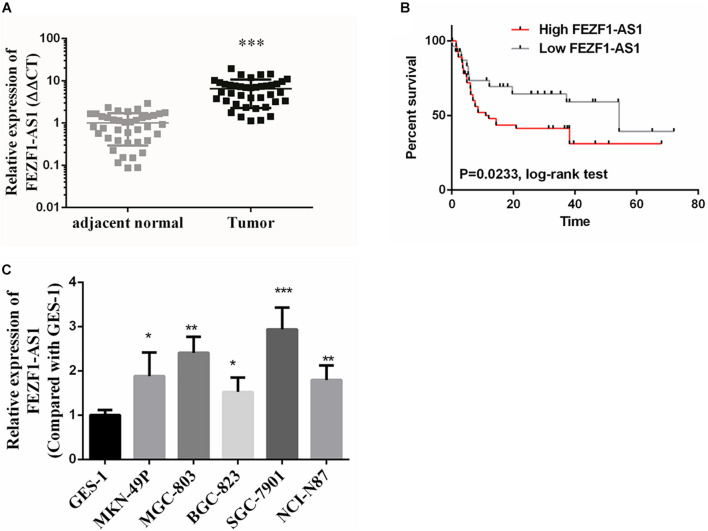
FEZF1-AS1 was upregulated in gastric cancer (GC) tissues and cell lines. **(A)** Relative expression of FEZF1-AS1 in 40 paired GC tissues and pair-matched adjacent normal tissues quantified by qRT-PCR. FEZF1-AS1 was upregulated (>2-fold) in 70% (28 of 40) of the GC tissues compared with that in the adjacent normal tissues. **(B)** Kaplan–Meier survival analysis of patient overall survival according to FEZF1-AS1 levels in GC tissues. Patients with high level of FEZF1-AS1 expression demonstrated reduced overall survival compared with patients with low expression. **(C)** FEZF1-AS1 expression in several types of gastric cancer cell lines and a normal human gastric epithelial cell line (GES-1) by qRT-PCR. Each experiment was performed in triplicate. Data are mean ± SD. *P < 0.05, **P < 0.01, ***P < 0.001.

In this study, GC tissues (*n* = 40) and pair-matched adjacent normal tissues (*n* = 40) were collected, and the total RNA of these specimens were extracted with TRizol reagents. The relative expression of lncFEZF1-AS1 was analyzed by qRT-PCR and the results were normalized to β-actin. As shown in Figure 1A, the expression of FEZF1-AS1 was considerably up-regulated in GC tissues compared with the normal control tissues (*P* < 0.001). Additionally, the relationship between the levels of FEZF1-AS1 and clinicopathological features was analyzed. The results showed that the expression levels of FEZF1-AS1 were significantly associated with poor clinicopathological features of GC. GC patients were subdivided into high- and low- FEZF1-AS1 expression groups and it was found that there was a statistically inversed association between FEZF1-AS1 expression and overall survival (OS; *P* < 0.05) (Figure 1B). Moreover, the expression levels of FEZF1-AS1 were analyzed in several GC cell lines, including MKN-49P, MGC-803, BGC-823, SGC-7901, and NCI-N87 cells. The normal human gastric epithelial cell line (GES-1) was used as a control. The qRT-PCR results showed that the expression of FEZF1-AS1 was upregulated in all these GC cell lines compared with GES-1 cells (Figure 1C). These results suggest that upregulation of FEZF1-AS1 might play an important role in the pathogenesis of GC.

### Overexpression of LncFEZF1-AS1 Promoted the Proliferation of Gastric Cancer Cells

**FIGURE 2:**
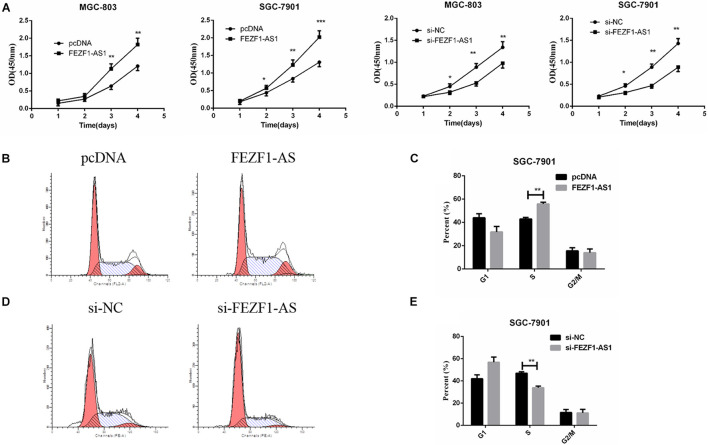
FEZF1-AS1 promoted GC cell proliferation *in vitro*. **(A)** Effects of FEZF1-AS1 overexpression or knockdown on GC cell proliferation measured by CCK-8 assay. **(B,C)** FACS analysis on the effects of FEZF1-AS1 overexpression on cell cycle in SGC-7901 cells. **(D,E)** FACS analysis on the effects of FEZF1-AS1 knockdown on cell cycle in SGC-7901 cells. The results represented the mean ± SD of 3 independent experiments. *P < 0.05, **P < 0.01, ***P < 0.001.

In order to elucidate the role of FEZF1-AS1 in GC cells, MGC-803 and SGC7901 cells with high expression of FEZF1-AS1 were selected for further studies. For overexpression of FEZF1-AS1, FEZF1-AS1 was cloned into pcDNA3.1 vector and transfected into MGC-803 and SGC 7901 cells. FEZF1-AS1-specific Si-FEZF1-AS1 RNAi was designed and transfected to knockdown the expression of FEZF1-AS1 in MGC-803 and SGC 7901 cells. Then the cell proliferation was analyzed by CCK8 assay and the results showed that overexpression of FEZF1-AS1 promoted the proliferation of MGC-803 and SGC 7901 cells while knockdown FEZF1-AS1 with si-FEZF1-AS1 markedly inhibited the proliferation of both MGC-803 and SGC 7901 cells (Figure 2A).

Moreover, cell cycle was analyzed by flow cytometry after overexpression or knockdown FEZF1-AS1 in GC cells. The results revealed that FEZF1-AS1 overexpression increased the proportion of cells at S phases, indicating that cell cycle was accelerated in FEZF1-AS1 overexpressed SGC 7901 cells (Figures 2B,C). While knockdown the expression of FEZF1-AS1 in SGC 7901 cells decreased the proportion of cells at S phases, indicating that cell cycle of GC cells was inhibited when knockdown the expression of FEZF1-AS1 (Figures 2D,E). Additionally, the cell cycle checkpoint factors, p21 and cyclin D1 expression were regulated by FEZF1-AS1 (Supplementary Figure 1). These results suggest that FEZF1-AS1 promotes GC progression by accelerated cancer cell cycle.

### FEZF1-AS1 Was Upregulated in Chemo-Resistant Gastric Cancer Tissues and Knockdown of FEZF1-AS1 Improved Chemo-Sensitivity in Gastric Cancer Cells

**FIGURE 3:**
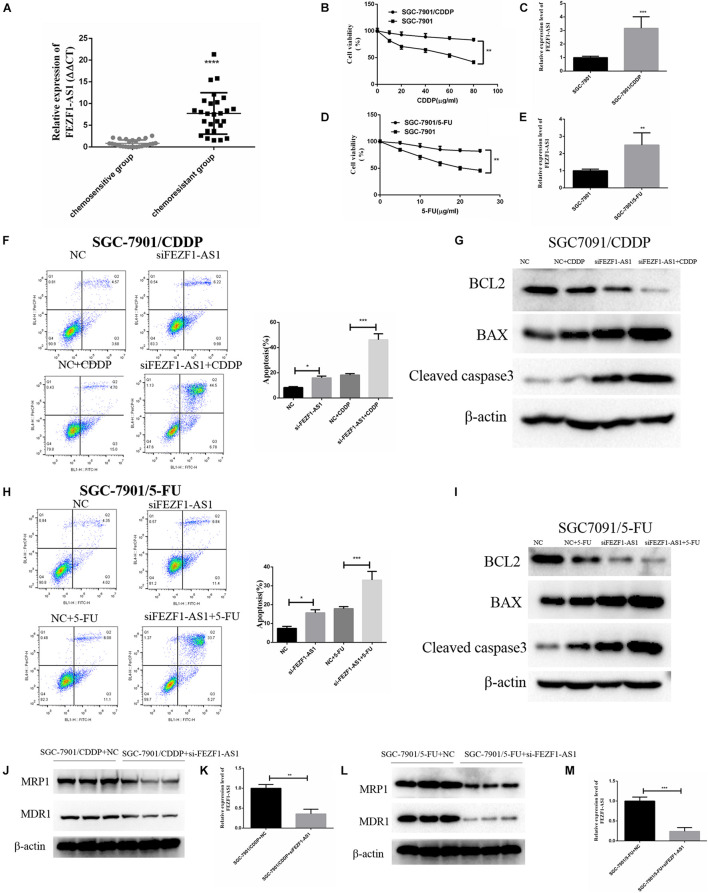
FEZF1-AS1 was upregulated in chemo-resistant GC tissues and knockdown of FEZF1-AS1 improved chemo-sensitivity of GC cells. **(A)** qRT-PCR on the level of FEZF1-AS1 in 27 pairs of specimens from GC patients with poor response to chemotherapy were 3.5-fold increase compared with patients with good response. **(B)** CCK8 assay on the viability of SGC-7901 and SGC-7901/CDDP cells treated with 10–80 μg/ml of CDDP for 24 h. **(C)** qRT-PCR on the level of FEZF1-AS1 in SGC-7901 and SGC-7901/CDDP cells. **(D)** CCK8 assay on the viability of SGC-7901 and SGC-7901/5-FU cells treated with 5–25 μg/ml of 5-FU for 24 h. **(E)** qRT-PCR on the level of FEZF1-AS1 in SGC-7901 and SGC-7901/5-FU cells. **(F)** Representative images and quantitation of flow cytometric analysis of apoptotic SGC-7901/CDDP cells with or without the treatment of FEZF1-AS1 siRNA after 24 h incubation with CDDP (40 mg/ml). **(G)** Western blot detected apoptotic proteins, BCL2, BAX, and Cleaved caspase3 in SGC-7901/CDDP cells with or without the treatment of FEZF1-AS1 siRNA after 24 h incubation with CDDP (40 μg/ml). **(H)** Representative images and quantitation of flow cytometric analysis of apoptotic SGC-7901/5-FU cells with or without the treatment of FEZF1-AS1 siRNA after 24 h incubation with 5-FU (20 μg/ml). **(I)** Western blot detected apoptotic proteins, BCL2, BAX, and Cleaved caspase3 in SGC-7901/5-FU cells with or without the treatment of FEZF1-AS1 siRNA after 24 h incubation with 5-FU (20 μg/ml). The protein expression of MDR1 and MRP1 in SGC-7901/CDDP **(J)** and SGC-7901/5-FU **(L)** cells detected by Western blot, FEZF1-AS1 siRNA decreased the protein levels of MDR1 and MRP1 in both cells. The knockdown efficiency of FEZF1-AS1 in SGC-7901/CDDP **(K)** and SGC-7901/5-FU **(M)** cells detected by qRT-PCR. Each experiment was performed in triplicate. Data are mean ± SD. *P < 0.05, **P < 0.01, ***P < 0.001, ****P < 0.0001.

The above results demonstrated that LncFEZF1-AS1 was overexpressed in GC tissues and correlated with poor prognosis of GC patients; overexpression of LncFEZF1-AS1 promoted the proliferation of GC cells, suggesting that LncFEZF1-AS1 might play a role in the development of GC. We further investigated whether FEZF1-AS1 plays a role in chemoresistance of GC. Chemo-resistant GC tissues (*n* = 27) and chemosensitivity GC tissues (*n* = 27) were collected, and the total RNA of these specimens was extracted with TRizol reagents. The relative expression of lncFEZF1-AS1 was analyzed by qRT-PCR. The results showed that the level of FEZF1-AS1 was considerably upregulated in chemo-resistant GC tissues compared to the chemosensitive GC tissues (Figure 3A), suggesting that high level of FEZF1-AS1 conferred drug resistance of GC cells. To investigate the effect of LncFEZF1-AS1 in the chemoresistance of GC cells, 5-FU-resistant SGC-7901 (named SGC-7901/5-FU) and cisplatin-resistant SGC-7901 (named SGC-7901/CDDP) were constructed and the 5-FU or CDDP resistance was measured with CCK8 assay. Compared with the control cells, it was observed that the cell viability of SGC-7901/CDDP cells increased significantly after treatment with 10–100 mg/ml of CDDP for 24 h (Figure 3B). The qRT-PCR result showed that FEZF1-AS1 was upregulated in CDDP-resistant SGC-7901 cells (Figure 3C). Moreover, it was observed that most of the SGC-7901/5-FU cells survived after treatment with 5–25 mg/ml of 5-FU for 24 h (Figure 3D). FEZF1-AS1 was also upregulated in 5-FU-resistant SGC-7901 cells (Figure 3E). These data suggested that SGC-7901/CDDP and SGC-7901/5-FU cells were resistant to CDDP and 5-FU, and FEZF1-AS1 was upregulated in chemo-resistant GC cells and tissues.

To further analyze the effect of FEZF1-AS1 on CDDP or 5-FU induced apoptosis in SGC-7901/CDDP cells or SGC-7901/5-FU cells, flow cytometric analysis was performed. After 40 g/ml CDDP treatment, apoptosis was significantly increased in the FEZF1-AS1 knockdown SGC-7901/CDDP cells (Figure 3F), and Western blotting also showed that the expression of apoptosis-related proteins (BCL2, BAX, and cleaved caspase3) was consistent with the apoptosis results in the FEZF1-AS1 knockdown SGC-7901/CDDP cells (Figure 3G). The apoptosis was also significantly promoted in the FEZF1-AS1 knockdown SGC-7901/5-FU cells when treated with 20 g/ml 5-FU (Figure 3H), the apoptosis biomarkers have similar expression to SGC-7901/CDDP cells (Figure 3I). The Western blot results showed that the protein levels of MDR1 and MRP1 were downregulated significantly after FEZF1-AS1 silencing in both SGC-7901/CDDP (Figures 3J,K) and 5-FU-resistant SGC-7901 cells (Figures 3L,M). These results demonstrated that FEZF1-AS1 was upregulated in chemo-resistant GC tissues and knockdown of FEZF1-AS1 improved chemo-sensitivity in GC cells.

### FEZF1-AS1 Regulated Chemo-Resistance of Gastric Cancer Cells Through Modulating Autophagy

**FIGURE 4:**
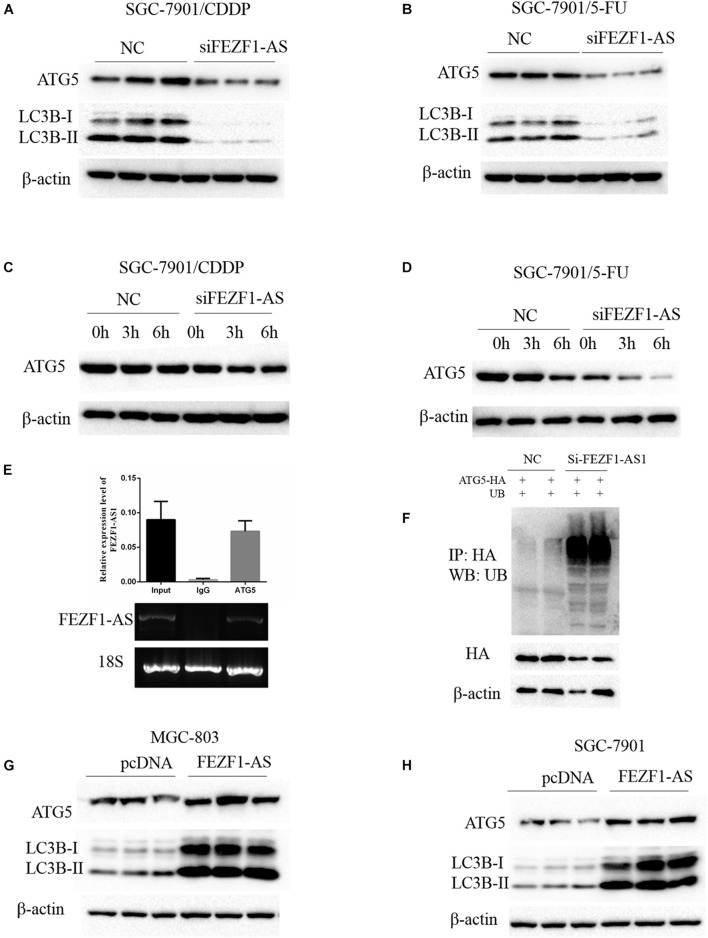
FEZF1-AS1 regulated chemo-resistance of GC cells through modulating autophagy. Western blot analysis of LC3 and ATG5 protein levels in SGC-7901/CDDP cells **(A)** and SGC-7901/5-FU cells **(B)** treatment with FEZF1-AS1 siRNA or NC. The protein stability of ATG5 in SGC-7901/CDDP cells **(C)** and SGC-7901/5-FU cells **(D)** analyzed by Western blot in cells treated with cycloheximide (50 μg/ml) at different time points. **(E)** RIP assays using an anti-ATG5 antibody showed that ATG5 interacts with FEZF1-AS1 in SGC-7901 cells. The qRT-PCR results of RIP assays are shown in the top. The results of agarose electrophoresis of the PCR products are shown in the bottom. **(F)** Knockdown the expression of FEZF1-AS1 increased the ubiquitous modification of ATG5. Western blot analysis of LC3 and ATG5 protein levels in MGC-803 **(G)** cells and SGC-7901 **(H)** cells transfected with FEZF1-AS1 overexpression plasmids or its vector plasmids. Each experiment was performed in triplicate.

Previous studies reported that autophagy is one of the important mechanisms of increased chemo-resistance in tumor cells including GC cells (Li et al., 2019). Whether FEZF1-AS1 regulated chemo-resistance of GC cells through modulating autophagy was not known. Therefore, we investigated the role of FEZF1-AS1 in autophagy and chemosensitivity in GC cells. The Western blot results showed that the protein levels of ATG5 and LC3-II were decreased significantly when knockdown of FEZF1-AS1 in SGC-7901/CDDP cells (Figure 4A), indicating that silence of FEZF1-AS1 inhibited autophagy in SGC-7901/CDDP cells. Similar phenomenon was observed in SGC-7901/5-FU cells with knockdown of FEZF1-AS1 (Figure 4B). Furthermore, the protein stability of ATG5 in SGC-7901/CDDP and SGC-7901/5-FU cell was analyzed by Western blot after the cells were treated with Cycloheximide (50 μg/ml) at different time points. It was found that knock down of FEZF1-AS1 by FEZF1-AS1 siRNA markedly decreased the protein stability of ATG5 both in SGC-7901/CDDP cells (Figure 4C) and SGC-7901/5-FU cells (Figure 4D). Moreover, we investigated the possible mechanism of FEZF1-AS1 in modulating the protein stability of ATG5. RIP assay was performed, the results showed that the ATG5 protein and FEZF1-AS1 was existed in the same complex (Figure 4E), suggesting the possible direct interaction between ATG5 and FEZF1-AS1. In addition, ubiquitination degradation of ATG5 was analyzed by an *in vivo* ubiquitination assay, the results showed that knock down of FEZF1-AS1 by FEZF1-AS1 siRNA markedly increased ubiquitination degradation of ATG5 in SGC-7901/CDDP cells (Figure 4F). The effects of overexpression of FEZF1-AS1 in MGC-803 and SGC 7901 cells were also analyzed by Western blot. The results showed the protein levels of ATG5 and LC3-II were increased significantly when overexpression of FEZF1-AS1 in MGC-803 (Figure 4G) and SGC 7901 cells (Figure 4H). These results suggested that FEZF1-AS1 regulated chemo-resistance of GC cells through modulating autophagy.

### The Proliferation and Autophagy of Gastric Cancer Cells Promoted by Overexpression of LncFEZF1-AS1 Were Suppressed by ATG5 Knockdown

**FIGURE 5:**
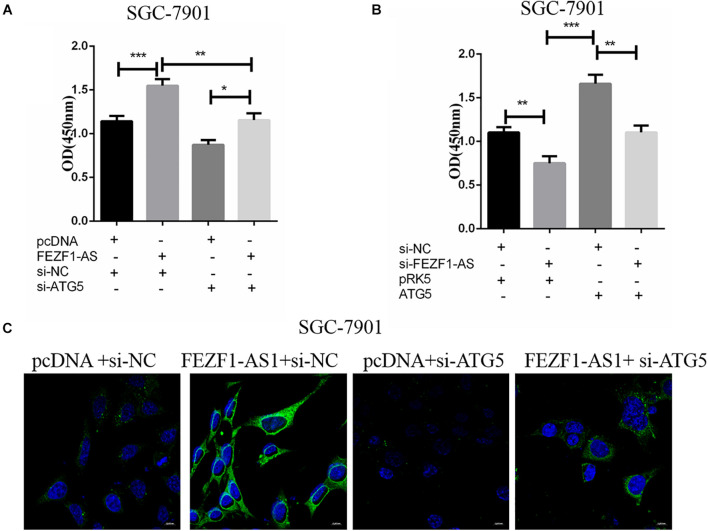
ATG5 knockdown inhibited autophagy of GC cells promoted by overexpression of LncFEZF1-AS1. **(A)** CCK8 assay on the proliferation of SGC-7901 cells with or without the treatment of FEZF1-AS1, FEZF1-AS1 + ATG5 siRNA or their control. **(B)** The proliferation of SGC-7901 with or without the treatment of FEZF1-AS1 siRNA, FEZF1-AS1 siRNA + ATG5 or their control group analyzed by CCK8 assay. **(C)** Representative images of LC3 puncta formation in SGC-7901 observed via IF staining, green: LC3B, bule: DAPI, scar bar: 20 mM. The results represented the mean ± SD of 3 independent experiments. *P < 0.05, **P < 0.01, ***P < 0.001.

In order to further investigate the role of ATG5 in FEZF1-AS1 in modulating proliferation of GC cells. SGC-7901 GC cells were transfected with FEZF1-AS1 overexpressing plasmids and vector control plasmids or co-transfected with si-ATG5 or si-NC. Then CCK8 assays were performed to analyze the proliferation of SGC-7901 GC cells. The results showed that there was no significant difference in cell proliferation in cells overexpressed LncFEZF1-AS1 plus si-ATG5 compared to cells with si-ATG5, indicating that cell proliferation promoted by FEZF1-AS1 was inhibited after knockdown of ATG5 in SGC-7901 GC cells (Figure 5A). Moreover, we investigated whether overexpression of ATG5 could rescue the proliferation of GC cells inhibited by FEZF1-AS1 knockdown in SGC-7901 GC cells. Four groups of SGC-7901 were set as follows: si-NC plus PRK5 vector, si-FEZF1-AS1 plus PRK5 vector, ATG5 plus si-NC and si-FEZF1-AS1 plus ATG5, then the proliferation of SGC-7901 was analyzed by CCK8 assay. The results showed that overexpression of ATG5 rescued the proliferation of GC cells inhibited by FEZF1-AS1 knockdown in SGC-7901 GC cells (Figure 5B). Furthermore, to further verify the effect of FEZF1-AS1 overexpression and ATG5 siRNA on autophagy of SGC-7901, LC3 puncta formation was observed via LC3B immunofluorescence staining. The results showed that FEZF1-AS1 overexpression increased the level of LC3 puncta markedly in SGC-7901 cells (Figure 5C), while ATG5 siRNA greatly inhibited puncta formation induced by overexpression of FEZF1-AS1. These results suggested that knockdown of ATG5 markedly inhibited the proliferation and autophagy of GC cells promoted by overexpression of LncFEZF1-AS1.

### Knockdown of FEZF1-AS1 Inhibited Tumor Growth and Increased 5-FU Sensitivity in Gastric Cancer Cells *in vivo*

**FIGURE 6:**
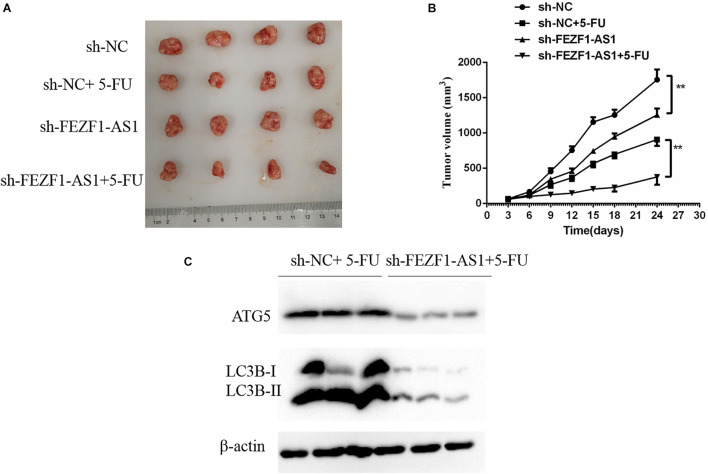
Knockdown of FEZF1-AS1 increased 5-FU sensitivity in tumors *in vivo*. **(A)** Representative images of xenograft tumors. **(B)** Tumor volume of mice. **(C)** Western blot analysis of LC3 and ATG5 protein levels in sh-NC-SGC7901 and sh-FEZF1-AS1-SGC7901 mice treated with 5-FU. Data were presented as the means ± SD from 4 tumor samples. **P < 0.01.

The above results suggested that high level of FEZF1-AS1 increased proliferation and multi-drug resistance of GC cells *in vitro*. Further studies were conducted to analyze whether FEZF1-AS1 plays the same effect *in vivo*. In order to investigate the possible functional role of FEZF1-AS1 in 5-FU resistance of GC cells *in vivo*, the stable sh-NC-SGC7901 and sh-FEZF1-AS1-SGC7901 cells were constructed through lentivirus infection and selected with puromycin. Then the cells were subcutaneously injected into nude mice to generate a xenograft tumor model, and the xenograft mice were treated with 5-FU or vehicle. The tumor volume of mice was measured and tumor growth curves were drawn. The results showed that the tumor growth was dramatically slower in the mice of the sh-FEZF1-AS1-SGC7901 group compared to the sh-NC-SGC7901 group, as evidenced by the reduction of tumor volume (Figures 6A,B). Additionally, knockdown of FEZF1-AS1 dramatically increased 5-FU sensitivity of GC cells *in vivo* as evidenced by the tumor growth was dramatically slower in the mice of the sh-FEZF1-AS1-SGC7901 group compared to the sh-NC-SGC7901 group after 5-FU treatment (Figures 6A,B). Western blot was performed to detect the protein levels of ATG5 and LC3-II in tumor tissues. It was found that the protein levels of ATG5 and LC3-II in tumor tissues from the sh-FEZF1-AS1-SGC7901 group was much lower than those from the sh-NC-SGC7901 group when treated with 5-FU (Figure 6C). These results indicate that knockdown of FEZF1-AS1 inhibited tumor growth and increased 5-FU sensitivity in GC cells *in vivo*.

## Discussion

Gastric cancer is one of the most common malignant diseases worldwide and its complex molecular mechanisms lead to a large number of GC-related deaths every year (Machlowska et al., 2020; Sexton et al., 2020). Although recent developments in diagnostics and therapeutics in GC treatment have improved the clinical outcome, effective treatment is still limited in a number of GC patients (Sasako, 2020; Sexton et al., 2020). The differentiation, proliferation, metastasis and chemoresistance of GC cells leads to unsuccessful treatment of GC in clinic (Machlowska et al., 2020; Sexton et al., 2020; Tyczynska et al., 2021). Therefore, understanding the molecular mechanisms of GC, particularly the mechanisms of chemoresistance could provide effective treatment for GC.

Long non-coding RNAs (lncRNAs) are important regulatory molecules that regulate gene expression via various aspects, including chromatin modification, transcriptional and posttranscriptional processing and so on (Yu and Rong, 2018). Recently, studies have revealed that lncRNAs play critical roles in the carcinogenesis and cancer progression of a variety of cancers, including GC (Yu and Rong, 2018; Ghafouri-Fard and Taheri, 2020). For example, FEZF1-AS1, a top overexpressed lncRNA in colorectal cancer, has been found to be involved in the initiation and progression of various cancers including colorectal carcinoma (Chen et al., 2016; Bian et al., 2018), non-small cell lung cancer (Huang et al., 2020) and gastric cancer (Wu et al., 2017). However, the molecular mechanism of FEZF1-AS1 plays in GC, particularly in chemoresistance of GC cells has not yet been clarified.

In the present study, our results showed that FEZF1-AS1 was up-regulated in GC tissues and cells. Additionally, high level of FEZF1-AS1 was significant correlated with poor prognosis in GC. Notably, overexpression of FEZF1-AS1 promoted the proliferation of GC cells. The cell cycle was inhibited when the FEZF1-AS1 was silenced in GC cells. These findings were consistent with previous reports (Wu et al., 2017; Hui et al., 2020). However, no study had reported the role of FEZF1-AS1 in chemoresistance of GC cells. Several previous studies had reported lncRNAs are emerging as new and valuable molecules that are involved in tumorigenesis and chemotherapy resistance of various tumors including GC (Fang et al., 2016; Yuan et al., 2016; Zhang et al., 2017). In this study, we observed that FEZF1-AS1 was upregulated in chemo-resistant GC tissues. Therefore, we further investigated the involvement of FEZF1-AS1 in chemo-resistance of GC.

Our results showed that FEZF1-AS1 was upregulated both in chemo-resistant GC tissues and chemo-resistant GC cells *in vitro*. Knockdown of FEZF1-AS1 significantly increased chemo-sensitivity of GC cells as evidenced by CDDP or 5-FU induced apoptosis in SGC-7901/CDDP cells or SGC-7901/5-FU cells after FEZF1-AS1 Knockdown. Additionally, the Western blot results showed that the protein levels of MDR1 and MRP1 were downregulated significantly after FEZF1-AS1 silencing in both SGC-7901/CDDP cells and SGC-7901/5-FU cells. These results demonstrated that FEZF1-AS1 was upregulated in chemo-resistant GC tissues and knockdown of FEZF1-AS1 improved chemo-sensitivity in GC cells.

Then, we further investigated the possible mechanism underlying the regulative effect of FEZF1-AS1 on chemoresistance of GC cells. Several previous studies had reported that enhanced autophagy is one of the important mechanisms of increased chemo-resistance in several tumor cells including GC cells (Yuan et al., 2016; Pei et al., 2018; Yeon et al., 2021). Therefore, we hypothesized that FEZF1-AS1 can regulate chemoresistance in GC cells via modulating autophagy. Loss-of-function experiments revealed that FEZF1-AS1 knockdown decreased the protein levels of ATG5 and LC3, suggesting that the inhibition of FEZF1-AS1 knockdown on autophagy of GC cells. Furthermore, knock down of FEZF1-AS1 by FEZF1-AS1 siRNA markedly decreased the protein stability of ATG5 in both SGC-7901/CDDP cells and SGC-7901/5-FU cells, which is consistence with previous report (Wang et al., 2020). RIP assay indicated the possible direct interaction between ATG5 and FEZF1-AS1 and knock down of FEZF1-AS1 by FEZF1-AS1 siRNA markedly increased ubiquitination degradation of ATG5 in SGC-7901/CDDP cells. However, how FEZF1-AS1 regulate the ubiquitination of ATG5 still need further study. All of these results suggested that FEZF1-AS1 regulated chemo-resistance of GC cells perhaps through modulating autophagy. Meanwhile, our results showed that knockdown of ATG5 markedly inhibited the proliferation and autophagy of GC cells promoted by overexpression of LncFEZF1-AS1. Knockdown of FEZF1-AS1 inhibited tumor growth and increased 5-FU sensitivity in GC cells *in vivo.*

In summary, we found that LncFEZF1-AS1 was overexpressed in GC tissues and cell lines, and positively correlated with poor prognosis of GC patients. Overexpression of LncFEZF1-AS1 promoted the proliferation of GC cells. Furthermore, FEZF1-AS1 was upregulated in chemo-resistant GC tissues and knockdown of FEZF1-AS1 improved chemo-sensitivity in GC cells. FEZF1-AS1 regulated chemo-resistance of GC cells perhaps through modulating autophagy and knockdown of FEZF1-AS1 inhibited tumor growth and increased 5-FU sensitivity in GC cells *in vivo*. Our results provide a novel insight and possible therapeutic target for chemo-resistance of GC.

## Data Availability Statement

The original contributions presented in the study are included in the article/Supplementary Material, further inquiries can be directed to the corresponding authors.

## Ethics Statement

The studies involving human participants were reviewed and approved by the Ethics Committees of Affiliated Jiangyin Hospital of Southeast University Medical College. The patients/participants provided their written informed consent to participate in this study. The animal study was reviewed and approved by the ethics committees of Affiliated Jiangyin Hospital of Southeast University Medical College.

## Author Contributions

YK and WZ concepted this study. ZG, ZZ, and QS performed mice and cell experiments. ZG, ZZ, QS, GS, and JH performed molecular and biochemistry experiments. ZG, ZZ, YK, and WZ analyzed and collected data. YK and WZ prepared draft. All authors revised and agreed the final version of the manuscript.

## Conflict of Interest

The authors declare that the research was conducted in the absence of any commercial or financial relationships that could be construed as a potential conflict of interest.

## Publisher’s Note

All claims expressed in this article are solely those of the authors and do not necessarily represent those of their affiliated organizations, or those of the publisher, the editors and the reviewers. Any product that may be evaluated in this article, or claim that may be made by its manufacturer, is not guaranteed or endorsed by the publisher.

## Supplementary Material

Supplementary Figure 1 Effect of disruption of FEZF1-AS1 on cell cycle related genes and ATG5 mRNA levels. qRT-PCR detected the mRNA levels of cell cycle related genes (p21 and cyclin D1) and ATG5 in overexpressed **(A)** or knockdown **(B)** FEZF1-AS1 in SGC7901 cells. The results represented the mean ± SD of 3 independent experiments. ^∗^*P* < 0.05, ^∗∗^*P* < 0.01, ^∗∗∗^*P* < 0.001, n.s no significance.
