# Supplementary figures and images for: LncRNA FEZF1-AS1 Promotes Multi-Drug Resistance of Gastric Cancer Cells via Upregulating ATG5
Source: Front Cell Dev Biol. 2021 Nov 1;9:749129. doi: 10.3389/fcell.2021.749129 (PMC8591218; doi:10.3389/fcell.2021.749129)

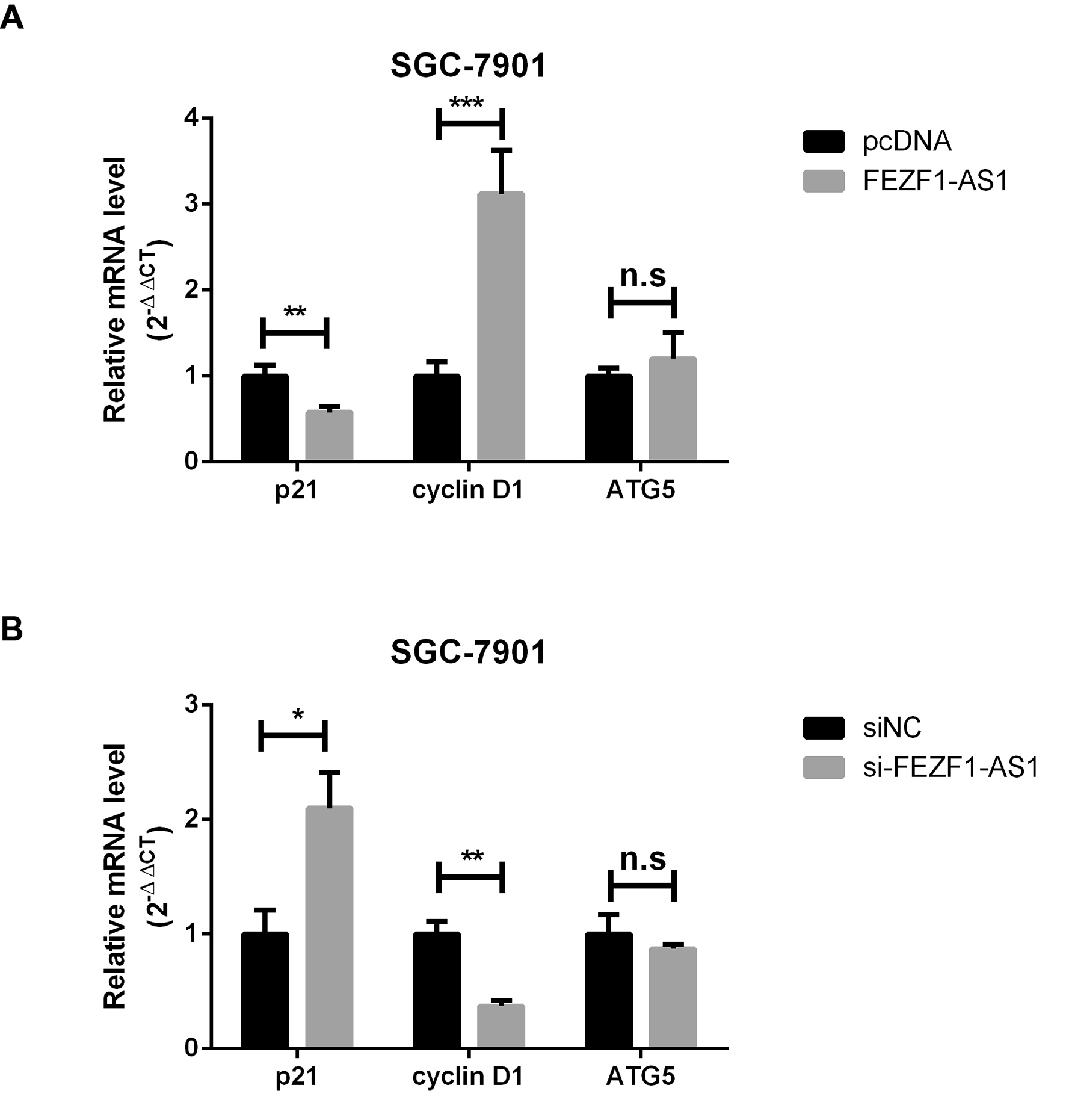

Supplement: Supplementary Figure 1 — Effect of disruption of FEZF1-AS1 on cell cycle related genes and ATG5 mRNA levels. qRT-PCR detected the mRNA levels of cell cycle related genes (p21 and cyclin D1) and ATG5 in overexpressed (A) or knockdown (B) FEZF1-AS1 in SGC7901 cells. The results represented the mean ± SD of 3 independent experiments. ∗P < 0.05, ∗∗P < 0.01, ∗∗∗P < 0.001, n.s no significance. [file Image_1.TIF]
